# The iMHere 2.0 System for Family Caregivers of Older Adults: A Focus Group

**DOI:** 10.5195/ijt.2023.6557

**Published:** 2023-05-11

**Authors:** Haomin Hu, Zara Ambadar, Eleanor Quinby, Yong K. Choi, I Made Agus Setiawan, Andi Saptono, Bambang Parmanto, Brad E. Dicianno

**Affiliations:** 1 Department of Health Information Management, School of Health and Rehabilitation Sciences, University of Pittsburgh, Pittsburgh, Pennsylvania, USA; 2 Department of Physical Medicine and Rehabilitation, School of Medicine, University of Pittsburgh, Pittsburgh, Pennsylvania, USA; 3 Department of Computer Science, Udayana University, Badung, Bali, Indonesia; 4 Human Engineering Research Laboratories, VA Pittsburgh Healthcare System, Pittsburgh, Pennsylvania, USA

**Keywords:** Community, Family caregivers, Home care, Older adults, Smartphone, Telerehabilitation

## Abstract

**Background:**

Family caregivers with continuous caregiving responsibilities are at increased risk for adverse physical and mental health outcomes. In response to the challenges of caregiving, a mobile health system (iMHere 2.0) was developed to support caregivers. The study's objective was to gather feedback from family caregivers of older adults on the current features of iMHere 2.0 and to formulate design criteria for future iterations of the system.

**Methods:**

An exploratory qualitative study with thematic analyses of focus group feedback.

**Findings:**

A total of 10 caregivers of older adults participated in a focus group. Five themes emerged: (1) *Monitoring health data*, (2) *Setting up customized reminders*, (3) *Supporting care coordination*, (4) *Balancing security and multiple user access*, and (5) *Disseminating iMHere 2.0 into the community*, along with some potential barriers to implementation.

**Conclusions:**

Design criteria were developed to provide a framework for iterative design and development of the iMHere system to support caregivers of older adults.

Family members often provide caregiving services to relatives with impairments or chronic conditions that limit one or more life activities (Schulz et al., 2020). The number of family caregivers continues to increase. In 2020, 53 million Americans provided care to an adult relative, and nearly 42 million (79.2%) were caregivers of recipients 50 years or older (AARP & NAC, 2020). Many factors prompted a shift toward family caregiving, including a critical shortage of professional caregivers and the effects from the COVID-19 pandemic (Kent et al., 2020).

As the care recipient ages, the need for family caregiving services increases, and the duration, intensity, and complexity of care needed also increases over time (Schulz et al., 2020). Taking care of loved ones requires family caregivers to monitor relatives' medical activities, support daily living, and coordinate with hired caregivers. Family caregivers must perform these functions while fulfilling necessary obligations and roles in their own lives. However, family caregivers may lack the same skills or training as professional caregivers. This may lead to an increased risk for adverse outcomes for caregivers who provide continuous care, including psychological distress, negative physical health outcomes, and problems with social relationships (Schulz et al., 2016). Unfortunately, high caregiver stress can also lead to the mistreatment of care recipients (Orfila et al., 2018). Thus, there is an urgent need to develop novel interventions to address the caregiving crisis.

Mobile health (mHealth) systems have been identified as effective ways to support family caregivers of older adults for self-management and delivery of home care and community services (Choi et al., 2014; Gellis et al., 2012). One systematic review on telehealth and caregivers indicated that technology-enabled interventions could support caregiving activities and promote the wellness of caregivers from the domains of education, consultation, cognitive behavior therapy, monitoring, clinical care delivery, and social support (Chi & Demiris, 2015). Also, mHealth systems were found to have the potential to enable timely communication and care coordination during the COVID-19 pandemic (Hajjar & Kragen, 2021). The flexibilities around telehealth provided opportunities make family caregiving easier (ACL, 2022).

Multi-component apps have had exponential growth in the mobile app market (Dicianno et al., 2015). Our previous studies used a user-centered approach to develop an adaptive mHealth system, “iMHere 2.0,” to support the self-management of people with chronic conditions (Bendixen et al., 2017; Setiawan et al., 2019). The system includes (1) a “client” app designed to be used by care recipients for self-management, (2) a “caregiver” app allowing caregivers to monitor the client's self-management activities, and (3) a “service provider” web portal for clinicians or service coordinators to manage teams of clients and caregivers and link them to services. Modules in the system enable self-management from the aspects of physical health, mental health, goal setting, personal health record storage, daily living tasks, and community integration. The use of the iMHere 2.0 system has been shown to be feasible in various service delivery models, including medical and rehabilitation care (Dicianno et al., 2016; Kryger et al., 2019) and community-based organizations (Ward et al., 2022). This paper aims to gain insight from family caregivers of older adults with chronic conditions on the potential use of iMHere 2.0 to support family caregiving. Specifically, we sought to use ideas from family caregivers to develop design criteria for future iterations of the system.

## Method

This is an exploratory qualitative study with thematic analyses of focus group feedback. The study was approved by the University of Pittsburgh's Institutional Review Board. Informed consent was obtained from all participants. The focus group was designed for individuals who were providing caregiving services to care recipients served by Allegheny County Area Agency on Aging (AAA). Allegheny County AAA is an agency that assists residents 60 years of age and older to live safe, healthy, and, when possible, independent lives. The focus group occurred after a large meeting for family caregivers of older adults organized by the county's AAA as part of their caregiver support program.

### Participants

Any interested family caregivers attending an Allegheny County AAA meeting were invited to attend the focus group. The inclusion criterion for the focus group was that participants must be family caregivers of older adults served by the AAA.

### Design

The focus group began with a brief presentation of the current iMHere 2.0 system, followed by focused discussion to gather feedback and ideas from participants on caregiving functions to be included in the future version. One research team member (BP) facilitated the presentation that demonstrated all features and modules of the system. The presentation emphasized the system's adaptiveness, reminder mechanisms, and modules related to daily living needs from the perspectives of both the care recipients and the caregivers. Throughout the presentation, the facilitator asked questions about the usability design of the system to understand whether the participants would accept the application of technology for their care service and whether the participants would become engaged with the system.

### Data Analysis

Participants' demographics, including age, gender, and race, were collected to characterize the sample. The proceedings of the focus group were audio-recorded and later transcribed. Participants' comments and feedback were formatted into units ranging from a brief six-word sentence to a paragraph of two sentences. Open and in-vivo coding was applied to identify new themes (Strauss & Corbin, 1990).

## Results

### Participants

A total of 10 family caregivers participated in the focus group. Participant demographics are shown in Table 1. Participants described the persons for whom they provided care as older adults with the following secondary diagnoses: dementia, lung cancer, chronic obstructive pulmonary disease, cerebral palsy, and diabetes.

**Table 1:** Participant Characteristics (n = 10)

Mean Age (SD)	65.3 (14.7) years
**Gender (n (%))**	
Male	4 (40%)
Female	6 (60%)
**Race (n (%))**	
White/Caucasian	8 (80%)
Asian-American	1 (10%)
African American	1 (10%)

### Feedback and Design Criteria

Participants felt that the iMHere 2.0 system may help support family caregiver services for older adults. Five overarching themes emerged from the data, including (1) *Monitoring health data*, (2) *Setting up customized reminders*, (3) *Supporting care coordination*, (4) *Balancing security and multiple user access*, and (5) *Disseminating iMHere 2.0 into the community*.

#### Theme 1: Monitoring Health Data

The *Monitoring health data* theme covered the significance of digitally tracking data and what data should be tracked. Participants felt that iMHere 2.0 had features for family caregivers to organize and track care recipients' information in one place. One participant stated:

*“I have some information currently on my iPhone, like the medicine she takes, the doctors she sees, those kinds of schedules and all. And I have other things written down, I would prefer to put all of that in one place that would enable another caregiver to see it.”*

Participants indicated that they kept notes on paper and within note-taking apps on their smartphones, electronic spreadsheets, and calendars, all of which were inconvenient and could not be shared quickly or securely. They pointed out that modules in iMHere 2.0 would allow family caregivers to keep track of care recipients' medication lists, appointment schedules, skincare issues, mood status, participation in exercise, and nutritional intake. In terms of existing features, one participant felt that the skincare module would help her to address her husband's skin problems. Participants suggested changes to the existing structure of the nutrition module. They were interested in tracking the percentage of a meal eaten instead of number of food groups consumed. They also requested features for tracking weight, vital signs (including pulse oximeter readings), insulin administration, and blood sugar levels. For example, one participant mentioned:

*“Do you keep track like weight on here? Or blood pressure, oxygen? Because somebody who might have congestive heart failure would gain weight overnight like 4 pounds or something. I think these are all important things because they are body functions that should be taken and taken every time you go to a doctor.”*

The *Monitoring health data* theme also included the concept of making the system user-friendly. Participants noted that they were concerned about data input frequency and complexity. They mentioned that, although the iMHere 2.0 system was portable and provided a variety of monitoring features, it may be challenging to use frequently while doing daily caregiving tasks. There could be a learning curve to get familiar with the system, especially if there is a lot of data to enter. One participant pointed out that:

*“I don't know that, honestly, I would have the time to keep this updated. I understand what you are saying, medication you are putting them in one time if the medication is not changing but I still need to daily go in there and say whether the medication somebody took it or not.”*

Most caregivers in the study were older adults caring for other older adults. They were concerned about their own technology literacy. Some felt they were not good at using technology, but the usability of the system may depend on the users' technology literacy. Additional requests from the participants included enabling voice input or connecting iMHere 2.0 system with intelligent agents (e.g., Siri, Alexa, Google) to enable voice control.

#### Theme 2: Setting Up Customized Reminders

The *setting up customized reminders* theme illustrates family caregivers' views on having reminders and alarms set up for caregiving needs. Family caregivers emphasized that they needed to ensure the accuracy of medical information while performing daily living tasks for their loved ones. They need to set up reminders, alarms, and schedules for these activities. One participant stated:

*“I had a relative that … because she thought she was gonna forget… she had alarm clocks in every room so when it was time to take her pill, or take her breathing treatment, the alarm clock went off to let her know.”*

Participants were impressed with the ability to set up customized reminders in iMHere 2.0. They felt that this would result in more accurate information that is less subject to recall bias, especially for medication administration.

iMHere 2.0 has a feature that allows care recipients to respond to reminders to take medications and note whether they took the medication in the Client app. Subsequently, a family caregiver can review the responses using the Caregiver app. Customized reminders help caregivers remind care recipients to take medications on time. Another benefit of these reminders requiring a response is that responses to the reminders could prevent care recipients with cognitive impairment from repeated intake of medications. As one participant stated:

*“Sometimes my sister has forgotten that she's taken one … She took it for a second time which caused her to have issues.”*

To avoid “reminder fatigue,” iMHere 2.0 is configurable to enable and disable or bundle reminders.

#### Theme 3: Supporting Care Coordination

The *supporting care coordination* theme describes the role that participants felt iMHere 2.0 could play in coordinating services among family caregivers and paid caregivers. Participants noted that the coordination of caregiving services is multi-faceted and often requires collaboration and communication. iMHere 2.0 has a feature that allows a care recipient using the Client app to be monitored by multiple caregivers using their Caregiver apps. One caregiver account can also be accessed through multiple devices. This feature provides flexibility for different caregiver roles, including: (1) one family caregiver having multiple devices, (2) family caregivers collaborating with hired caregivers, and (3) hired caregivers using the same shared account. iMHere 2.0 allows various caregivers to communicate about care and provide a safe handoff, especially when a new caregiver must replace a typical caregiver who is unavailable. One participant stated:

*“[This feature would be] beneficial to me and if I hire somebody to come in.”*

Another participant noted:

*“But I feel that if it's a caregiver situation where maybe you have the potential to have multiple individuals coming in and out of her home and providing care, it would be beneficial to have this, certainly to help with the communication and hand off and that would be helpful.”*

Participants identified the modules related to daily living tasks as a solution to keep track of hired caregivers' work. Family caregivers would benefit by reviewing the completion of assigned tasks delegated to hired caregivers, such as helping the care recipient with bathing, toileting, and dressing, when family caregivers are not at home.

#### Theme 4: Balancing Security and Multiple User Access

The *balancing security and multiple user access* theme discussed role-based accounts, access of multiple users, and passwords in iMHere 2.0 system. Participants acknowledged several advantages of the current configurations in iMHere 2.0. Role-based accounts distinguish family caregivers from hired caregivers. The discrete caregiver account types allow the care recipient to disclose only vital aspects of private information appropriate for each caregiver role, which prevents caregivers from accessing unnecessary private information. The ability to manage accounts remotely through the web portal was felt to be crucial to family caregiving. Participants stated that access should be managed separately on different caregivers' devices under an administrator role (usually the role of family caregivers), especially when a paid caregiver was off duty and another one came in. One participant was concerned:

*“… my sister has a caregiver…[but] she may have a different one …. Will that be two different passwords?”*

Participants were satisfied with the feature that requires a unique password or verification for each caregiver. They felt this feature would help prevent hired caregivers from modifying confidential data or continuing to access the system after termination of their caregiving role.

#### Theme 5: Disseminating iMHere 2.0 into the Community

The *disseminating iMHere 2.0 into the community* theme centered around the discussion of its potential use in long-term care services after research studies. Participants asked:

*“Do you have to purchase the app? Where would there be a fee?”*

*“Do you get this app by medical means or by purchasing it? Would it be through like the Medicare, would it be through insurance? Would it be through the university since it looks to me that the university is doing this research. So maybe it would be through the university? Through the insurance?”*

Although participants recognized the potential benefits of using iMHere 2.0, they also raised questions about its implementation and dissemination beyond research. Concerns included the fees associated with using iMHere 2.0, how it might be integrated into existing caregiver programs, and whether its use might be covered by insurance or other funding sources. Participants highlighted the importance of identifying a community service model for iMHere 2.0. Such a model could involve collaborations with healthcare providers, insurance companies, or government agencies to ensure that the technology is accessible and affordable.

A summary of design criteria based on the participants' feedback is shown in Table 2.

**Table 2:** Design Criteria for iMHere 2.0 for Family Caregivers

THEME	CATEGORY	REQUESTED FEATURES	REASONING
*Monitoring Data*	Trackers	Upgraded features for caloric and portion intake	Caregivers want to track either calories or portions of meals consumed
		Track weight	People who have certain conditions could have drastic weight gain or loss
		Track vital signs	Many older adults use portable oxygen equipment or have other blood pressure or oxygen level concerns
		Track insulin injection and blood sugar	People with diabetes could keep record of their blood sugar readings and insulin intake
	Usability	Fact sheet that allows quick entry of key data points	Would reduce burden of data entry and allow caregiver to quickly populate multiple modules at once. Especially helpful for caregivers who are not “tech savvy”
		More selections than typing	Would provide quick input
	Accessibility	Voice commands to control app and enter data	Eases control of app and reduces burden of data entry for caregiver
		Text to speech audio for reminders	Provides audio output of reminders to older adults (e.g., app reads aloud reminder to take a medication and the medication's purpose).
*Setting up Customized Reminders*	Mechanism	Remind-recall	Set up for various activities, remind on time, and track responses
	Device	Connect with Speakers	Audio output to ensure the delivery of reminders across rooms
*Supporting Care Coordination*	Flexibility	Communication and collaboration with multiple types of caregivers	Caregiving services is multi-faceted and often requires collaboration and communication
*Balancing Security and Multiple User Access*	Configuration	Role-based account	Access control
	Password	Users should have unique password/verification	Prevent invalid data modification and access
*Disseminating iMHere 2.0 into the community*	Implementation & Dissemination	Identify a service model to use iMHere 2.0 into community	Caregivers want to use iMHere 2.0 with benefits

## Discussion

### Principal Findings

This study adds to the literature by demonstrating how an mHealth system can support family caregivers of older adults. Our findings suggest that many aspects of the present iMHere 2.0 system would benefit family caregivers in taking care of their loved ones. The results align with the evidence that telehealth may support family caregivers engaged in home care and care recipients' self-management (Garnett et al., 2022; Graven et al., 2021).

This study highlights the importance of user-centered design and co-design approaches in the development of mHealth interventions for family caregivers of older adults (Sumner et al., 2021). The results of the study demonstrate that caregivers of older adults have unique needs and preferences for technology that will support them in their caregiving tasks.

The feedback and the design criteria provided by participants will help guide the ongoing development of iMHere 2.0 to meet the needs of family caregiving, reduce barriers to implementation and adoption, and provide a more robust user experience. Our iterative design process will include additional trackers and features to improve usability. Family caregivers themselves are at risk of adverse health outcomes (Schulz & Sherwood, 2008). Therefore, we are adding interventions to support caregivers' own health. We will leverage findings from interventions that have been found to be successful in supporting caregivers in (1) providing care, (2) relieving stress using meditation, (3) setting care goals and plans, and (4) coping (Schulz et al., 2003).

Our study findings recognize the importance of interoperability and highlight the potential benefits of integrating iMHere 2.0 with the electronic health record (EHR) and other connected devices, such as wearable sensors and intelligent agents. Data exchange between iMHere 2.0 and the EHR can streamline the data entry and reduce the user burden. For instance, by making user data available on a dashboard, clinicians can access the caregiver generated data at the point of care, together with information such as a list of current medications or details from the user's personal health record (PHR). Integrating smart connected devices offers several advantages, including passive monitoring, voice input, and smart voice control and user interactions. Passive monitoring enables caregivers to track data and monitor care recipients without requiring any active participation (Choi et al., 2019). Voice input makes it easier for caregivers to enter data into the system, especially for older adults who may have difficulty with typing or touchscreen navigation. Additionally, smart voice control allows caregivers to interact with the system using voice commands, providing a more natural and intuitive way of managing care tasks. The use of smart connected devices can help to improve the accuracy and reliability of the data collected, enhancing the quality of care provided to older adults.

Concerns from participants highlighted the critical role of technology literacy, usability, and accessibility in the successful implementation of iMHere 2.0 into the caregiving community. As most participants in the focus group were older adults themselves, they recognized that low technology literacy could be a significant barrier to adopting or using mHealth technologies like iMHere2.0. Therefore, it is important to balance the need for multiple features and the system's ease of use, ensuring that it is accessible and usable for older adults. To overcome technology literacy barriers, it may be necessary to provide training sessions and ongoing technical support to ensure that users feel confident and capable of using iMHere 2.0 effectively.

Another critical consideration for the successful implementation of iMHere 2.0 in communities is the need to identify a sustainable community service model. Without a sustainable service model, the cost of implementing and using iMHere 2.0 could be too high, making it inaccessible for many patients and caregivers. This could limit the technology's adoption and effectiveness in improving long-term care services.

Moreover, the COVID-19 pandemic has highlighted the challenges faced by family caregivers, making it more important than ever to find innovative solutions to support them. iMHere 2.0 has the potential to offer much-needed support and relief to family caregivers of older adults, allowing them to track their loved ones' health and receive timely alerts. By working to ensure the technology's accessibility and usability and by identifying a service delivery model that support the adoption and use of mHealth technologies in long-term care services, the technology can be made more widely available and effectively used by caregivers of older adults.

## Limitations

Several limitations of this study deserve discussion. First, the focus group met once, and the number of participants was small. Our design criteria may not represent the views of all potential family caregivers of older adults. For example, older adults are more likely to experience social isolation and related mental health problems. Participants in this study did not raise this issue. Social interactions are important in the caregiving context and associated with caregiving needs (Tang et al., 2019). Further feedback to establish design criteria for social support may be required. Second, iMHere 2.0 was initially created for self-management, with care recipients as its primary users. However, the majority of participants in this study believed that family caregivers should be the primary users of the system when the care recipient is an older adult. This feedback will inform future usability and accessibility studies to maximize use by family caregivers. Third, iMHere 2.0 was developed to support individuals in their homes and community settings. The focus group participants noted that the system may help in the process of transitioning from a hospital or skilled nursing facility to the home environment, but more work would be needed to create features that support such a transition.

## Implications

This study explored the potential use of iMHere 2.0 to support family caregiving. The findings suggest further research is needed to (1) implement design criteria and (2) evaluate the usability, accessibility, and feasibility of use of the system.

## Conclusions

This study demonstrated the potential for using iMHere 2.0 to support family caregiver services for older adults. Design criteria were developed to provide a framework for iterative design and development of this mHealth system.
